# Voges’ Description of Mal De Caderas, a South American Trypanosomatic Disease of Domestic Animals

**Published:** 1902-09

**Authors:** Ch. Wardell Stiles

**Affiliations:** Zoologist of Bureau of Animal Industry


### VOGES' DESCRIPTION OF MAL DE CADERAS, A SOUTH AMERICAN TRYPANOSOMATIC DISEASE OF DOMESTIC ANIMALS.

BY CH. WARDELL STILES, PH.D.,
ZOOLOGIST OF BUREAU OF ANIMAL INDUSTRY.

THE attention of the United States Government has recently been called to a serious malady of horses in the Philippines. This disease is known as surra, and is caused by a protozoon, *Trypanosoma Evansi* by name, which lives in the blood plasma and destroys the red blood corpuscles.

As soon as surra was diagnosed in the Philippines the Bureau of Animal Industry prepared an emergency report on the subject[1] discussing not only surra, but also allied maladies, such as tsetse-fly disease, dourine, and mal de caderas—three other equine mala-

[1] Salmon and Stiles. "Emergency Report on Surra, with a Bibliography of Allied Trypanosomatic Diseases, by Albert Hassall." Bulletin No. 42, Bureau of Animal Industry, United States Department of Agriculture, Washington, D. C., 1902, pp. 152, 112 illustrations.

dies described as being due to trypanosomatic parasites. At the time the report in question was written little was known regarding mal de caderas, but just after the proof-sheets were returned to press, and too late to make additions, an important paper on mal de caderas[1] appeared. This article contains many facts of interest to North American veterinarians and stock raisers, and on this account an extended review of Voges' paper is here given, so that mal de caderas may be compared with surra as described in the bulletin just mentioned.

The name *mal de caderas* is a vernacular term based upon one of the chief symptoms, namely, a paraplegia of the haunches, and means "disease of the haunches."

The disease appears to have existed for some years past in the central portion of South America. Although the first published reports date but a few years back (1899), an account of the malady was sent to Europe as early as 1842, but did not receive any attention. Voges divides the disease into two stages.

First stage: In experimental cases, if 5 to 20 c.c. of blood from a sick horse are injected into a healthy horse a fever develops four or five days later, increasing from hour to hour, and remitting somewhat on the following morning, or increasing steadily to 40° or 41° C. During the following day the temperature falls to or near the normal, usually reaching its lowest point on the second day. The temperature now rises again, day by day, until after another five days it again reaches or exceeds 40° C. This alternation occurs two, four, or even six or eight times in succession, the intermissions usually varying from three to six days. Pulse and respiration remain normal, and there is scarcely any change in the appetite except that in the evening, at the height of the fever, the appetite is slightly diminished; thirst increases. The feces are usually normal, but occasionally contain blood. Temporary hæmoglobinuria develops, and hæmaturia becomes almost constant. Sight and sense of hearing remain normal. The coat remains smooth and glistening, and in case the attack occurs during the period of the fall of the hair this process occurs in the normal manner. Reflexes are normal. The intermittent fever is the most characteristic of all the symptoms.

Second stage: The transition to the second stage is gradual. As the disease progresses the intermittent character disappears. The fever does not rise so high, 40° and 41° C. being exceptional;

[1] O. Voges. "Das Mal de Caderas." Zeitschrift f. Hygiene u. Infectionskrankheiten, Leipzig, vol. xxxix. (3), März 13, pp. 323–371, pl. 5, figs. 1–7. [Wa.]

the remissions are also less prominent. The temperature usually varies between 38.5° and 39.8° C. The animal lacks energy; the head hangs; the entire posture of the body becomes indolent, and even the wildest and meanest animals show no disposition to bite or kick. The patient becomes emaciated, although the appetite continues; thirst continues, and even increases.

Proportionally to the increasing general weakness of the body the heart becomes weaker, so that it cannot propel the blood; œdema develops, especially in the hind legs; the coat becomes staring. The sensibility is so decreased that the horse makes no effort to rid himself of the swarm of flies which settle upon him, but in some cases reflex movement remains unimpaired. Respiration, digestion, and feces remain normal or nearly so. Hæmoglobinuria is usually absent, but the urine nearly always contains red blood corpuscles. Urination is frequent and abundant, owing to the large amount of water taken in. The animal shows no signs of pain.

The gait is not always normal. In some cases the animals drag themselves slowly forward; in others their walk resembles that of a drunken man. The patients may fall, and then become so exhausted in their efforts to rise that they die within twenty-four hours. If they are "tailed up," however, they may continue to live eight to fourteen days longer; some animals are able to stand and walk to the last. Toward the end the temperature undergoes marked changes, varying from morning to evening between 34° and 39° C.; death rarely occurs during a high temperature. The entire illness may last from fourteen days to more than four months.

*Gross Pathology.* Staring coat and the above-described œdema are present. The hide is removed with difficulty, as the flesh is usually very dry, similar to the condition found in cholera patients. The pleural cavities contain a serous, yellow, usually clear or only slightly clouded exudate, often several litres in amount, and containing red and white blood corpuscles. Fibrous deposits are found on the pleuræ in small or in larger quantities. Lungs are normal. The cardiac sac is filled with a large amount of exudate similar to that found in the pleural cavity. The cardiac muscle is normal or dilated, and pale like the other muscles. The lymphatic glands are slightly swollen. The abdominal cavity contains an exudate similar to that found in the pleural cavity, and similar fibrinous deposits are found, especially upon the liver. The intestinal canal appears normal. The spleen is enormously enlarged, the enlargement being in proportion to the length of the attack. The liver also is enlarged. The kidneys are pale on section, and they, too, may be enlarged. The lymphatic glands are often swollen.

*Seasonal Conditions.* The disease occurs only in regions where swamps exist. It is also reported to be a wet-weather disease, and Dr. Kemmerich is quoted as authority for the statement that if the plants are removed from a swamp, and a permanent wave motion provided for, the disease is controlled. Kemmerich also advises that horses be kept in stables instead of in camps, and reports that this precaution resulted in stopping the disease.

In droves of horses in which the malady has broken out the disease may be arrested by taking the animals to a high, dry locality.

*Economic Importance.* Mal de caderas is of great importance from a military standpoint, as is seen from the fact that five cavalry regiments lost 1039 horses and 489 mules within six months, the deaths being due to this disease. The morbidity varies from 25 to 100 per cent. The lethality of mal de caderas is placed at 100 per cent.

*The Blood.* The blood count shows an enormous decrease in red blood corpuscles, from 10,000,000 down to 4,000,000 or 3,000,000, or even as low as 800,000; at the same time there is an increase in the white blood corpuscles. Subcutaneous injections of blood from diseased animals result in causing the disease, but experiments with the blood filtrate as well as with ingestion of blood were negative. The hæmoglobin decreases from 13.1 per cent. to 3 or 4 per cent.

*The Parasite.* Mal de caderas is a blood disease, like surra, caused by a parasitic protozoon, to which Voges in his preliminary paper gave the name *Trypanosoma equinum.* Voges' present description does not permit us to distinguish clearly between *Trypanosoma equinum* and certain allied species of the same genus. The reproduction of the parasite is described as consisting of (1) a longitudinal division, or (2) a transverse division, or (3) segmentation; but it seems probable that at least some of the cases of alleged transverse division represent the agglutination of the parasites. The organisms appear in the blood about five days after infection, increasing in proportion to the rise in temperature, until, as a rule, they suddenly disappear when the fever reaches 40° C. About three to five days later they can again be discovered, and they increase again in proportion to the animal's temperature. At their height they are about 10 to 25 per cent. as numerous as the red blood corpuscles. In the second stage of the disease this periodicity of the parasite becomes more or less obscure.

The important fact was determined that the blood is usually non-infectious twenty-four to forty-eight hours after death, thus showing a marked difference from bacterial diseases like anthrax.

*Animals Affected.* Asses and mules are much less susceptible to the disease than horses.

Mice—both the white and the gray—are especially susceptible. In twenty hours after the inoculation of white mice it is possible to find trypanosoma in the blood. Gray mice are less susceptible, and the parasites are not found until about three days after inoculation. In the case of white mice the parasites increase so rapidly that on the fourth or fifth day they are more numerous than the red blood corpuscles. Gray mice die about twelve to fourteen days after infection. All mice, without exception, die on the first attack. Thus mice are exceedingly susceptible to the disease, and are therefore very valuable as aids in diagnosis.

Rats are also susceptible. In the case of white rats the parasites appear in the blood of the tail four to five days after infection. Crosses between the white rat and the wander rat are more resistant, the protozoa not appearing in the blood until the fifth to the seventh day. As in the case of infection of mice, the parasites increase to such an extent in rats that the protozoa outnumber the red blood corpuscles. The wander rat is still more resistant, and in some cases the parasites disappear temporarily from the circulation. Most rats, however, die in the first attack.

Rabbits may live for one to three months after infection. The parasites may not be evident in the circulation for four weeks after inoculation. There is profuse lacrymation, the conjunctivæ become catarrhal, the eyes become dim, and a pus-like secretion forms, by which the lids are closed. In bucks the testicles swell and become inflamed, and similar changes appear in the vulva of doe rabbits.

Dogs contract the disease and live two to three months. The œdema is quite prominent, especially on the head and eyelids. The conjunctivæ become affected, and the eyes become dull and more or less inflamed—conditions which are similar to those which take place in rabbits. The œdematous condition of the scrotum is striking, but the penis is not affected. The dog is about as susceptible to mal de caderas as the rabbit or slightly less so. There are times when the parasite almost or quite disappears from the blood.

In sheep and goats the disease is fatal, but the animals may live for months without showing any special symptoms; then they become emaciated and suddenly die. The appearance of the parasites in the blood is periodical.

The disease was fatal to a monkey (*Nictipithecus felinus*).

Cats die about four weeks after infection without having shown any special symptoms during the disease.

Two specimens of *Nutria* were also killed with mal de caderas about ten days after infection.

Guinea-pigs are much less susceptible than the other animals, only about one-half to two-thirds of the infected animals dying. Death occurs from two to five months after infection.

Cattle appear to be immune, and Voges records a case where an animal was kept one and a half years, and, despite the fact that it was inoculated with large quantities of trypanosomatic blood, it gained in weight.

Mal de caderas is transmissible also to birds, and Voges inoculated chickens, ducks, and turkeys with fatal infections. Chickens die in the second or third week. During the disease they become very emaciated, but exhibit no other symptoms.

Voges' views relative to the cause of mal de caderas were opposed in South America, and in reply to his opponents he argues that *Trypanosoma equinum* of the horse cannot be identical with *Trypanosoma Lewisi* of rats. The disease is, however, similar to dourine and also similar to surra (Voges unites surra and nagana), but Voges considers mal de caderas distinct from both dourine and surra.

In reference to transmission, Voges has experimented with bloodsuckers, with negative results, and he now suspects flies belonging to the species *Musca brava.*

*Diagnosis.* The disease may be diagnosed with the aid of the microscope. Diagnosis of doubtful cases is made possible by injecting 1 or 2 c.c. of blood from the suspected horse into a mouse; the blood of the latter may then be examined after one to four days.

*Treatment.* Experiments in treatment with quinine, methylene blue, enterol, salicylate of soda, turpentine oil and permanganate of potash, iodide of potash, and intravenous injection of corrosive sublimate were all negative. Arsenious acid gave temporary favorable results. Experiments in serum therapy were negative.

